# Cementing does not increase the immediate postoperative risk of death after total hip arthroplasty or hemiarthroplasty: a hospital-based study of 10,677 patients

**DOI:** 10.1080/17453674.2019.1596576

**Published:** 2019-04-01

**Authors:** Elina Ekman, Inari Laaksonen, Kari Isotalo, Antti Liukas, Tero Vahlberg, Keijo Mäkelä

**Affiliations:** a Department of Orthopaedics and Traumatology, Turku University Hospital;;; b Department of Anaesthesiology and Intensive Care, University of Turku, Pohjola Hospital;;; c Department of Clinical Medicine, Biostatistics, University of Turku, Turku, Finland

## Abstract

Background and purpose — It has been suggested that cemented arthroplasty is associated with increased peri- and postoperative mortality due to bone cement implanting syndrome, especially in fracture surgery. We investigated such an association in elective total hip arthroplasty (THA) patients and hemiarthroplasty (HA) patients treated for femoral neck fracture.

Patients and methods — All 10,677 patients receiving elective THA or HA for fracture in our hospital between 2004 and 2015 were identified. Mortality rates for cemented and uncemented THA and HA were compared at different times postoperatively using logistic regression analysis. Analysis was adjusted for age, sex, ASA class, and year of surgery.

Results — Adjusted 10- and 30-day mortality after cemented THA was comparable to that of the uncemented THA (OR 1.7; 95% CI 0.3–8.7 and OR 1.6; CI 0.7–3.6, respectively). There was no statistically significant difference in the adjusted 2-day mortality in the cemented HA group when compared with the uncemented group. However, in a subgroup analyses of ASA-class IV HA patients there was a difference, statistically not significant, during the first 2 days postoperatively in the cemented HA group compared with the uncemented HA group (OR 2.1; CI 0.9–4.7).

Interpretation — Cementing may still be a safe option in both elective and hip fracture arthroplasty. Excess mortality of cemented THA and HA in the longer term is comorbidity related, not due to bone cement implantation syndrome. However, in the most fragile HA patient group caution is needed at the moment of cementing.

Total hip arthroplasty (THA) is considered a safe procedure and over recent years early postoperative mortality has decreased (Hunt et al. 2013). In a recent systematic review, mortality during the first 30 postoperative days after THA was 0.3% (Berstock et al. 2014).

Conversely, femoral neck fracture is associated with high peri- and postoperative mortality. 30-day mortality has been reported to be as high as 5–10% (Keating et al. 2006, Moja et al. 2012, Smith et al. 2014). This is largely explained by most of these patients being fragile with several comorbidities, such as cardiovascular diseases, cognitive impairment, and poor pre-fracture mobility.

THA can be performed as cemented, uncemented, hybrid (uncemented cup and cemented stem) or reverse hybrid (cemented cup and uncemented stem) whereas hemiarthroplasty can be performed with or without bone cement. Cemented THA has superior implant survival rates compared with uncemented THA in long term follow-up in elderly patients (Issack et al. 2003, Buckwalter et al. 2006, Morshed et al. 2007, Mäkelä et al. 2014). In addition, national guidelines in Finland, Sweden, and the UK recommend cemented HA in femoral neck fracture (National Clinical Guideline 2011, Rogmark et al. 2014, Current Care Guidelines 2017). However, some surgeons hesitate to use bone cement due to the possibility of bone cement implantation syndrome (BCIS), which may cause cardiovascular disturbances, pulmonary embolism, and at worst death of the patient (Donaldson et al. 2009).

We investigated whether the use of bone cement is associated with higher immediate mortality in patients treated with THA or HA in Turku University Hospital. We also assessed separately whether there is a difference in the early postoperative mortality in the most fragile (ASA-class IV–V) patients treated with cemented or uncemented HA.

## Patients and methods

### Source of data

This study was performed at Turku University Hospital, Turku, Finland. Patients operated for OA, rheumatoid arthritis, psoriatic arthritis, juvenile arthritis, unspecified arthritis, or femoral neck fracture (ICD-10 codes M16.0 - M16.9, M05.8, M05.9, M06.0, M07.3, M08.0, M08.3, M13.9, S72.0) with uncemented, cemented, or hybrid THA (ICD-10 codes NFB30, NFB40, and NFB50) or with cemented or uncemented HA (ICD-10 code NFB10, NFB20) were included in the study. During the study period from January 1, 2004 to May 8, 2015, 7,569 primary THAs and 3,108 HAs were performed in Turku University Hospital. For every patient the preoperative diagnosis and sex, age, and ASA class at the time of the operation were recorded. Time of death was obtained from the National Causes of Death Statistics maintained by Statistics Finland.

### Study population

Of the 7,569 primary THAs 74% were uncemented,18% cemented, and 8.5% hybrid. 60% of the THA operations were performed on women and the most common preoperative diagnosis was OA (75%). Of the HAs 38% were uncemented and 62% were cemented. During the study period the use of cemented THA decreased in Finland whereas the use of uncemented THA increased (Finnish Arthroplasty Register [FAR] 2017). The use of uncemented HA increased a little while the use of cemented HA remained constant (Yli-Kyyny et al. 2014). In the HA group 71% of the operations were performed on women and all these operations were performed due to femoral neck fracture. In all study groups, ASA classes I, IV, and V were highly uncommon and we therefore grouped ASA classes I–II and IV–V together. The baseline characteristics for the study groups are given in Table 1.

**Table 1.** Baseline characteristics for the patient cohorts

	Uncemented	Cemented	Uncemented	Hybrid	Cemented
	HA	HA	THA	THA	THA
Number of patients	1,142	1,868	4,855	612	1,274
Number of operations	1,173	1,935	5,563	640	1,366
Percentage of females	67	74	56	68	73
Mean age (SD)	81 (10)	83 (8)	65 (10)	75 (8)	77 (6)
range	37–107	40–103	13–96	38–91	42–97
ASA class 1–2, n (%)	115 (10)	194 (10)	3,303 (59)	258 (40)	494 (36)
ASA class 3, n (%)	707 (60)	1,234 (64)	2,163 (39)	363 (57)	819 (60)
ASA class 4, n (%)	349 (30)	501 (26)	86 (2)	18 (3)	52 (4)

THA = total hip arthroplasty. HA = hemiarthroplasty.

A third-generation cementing technique (washing the bone with pulsed lavage, use of an intramedullary plug and retrograde insertion of the cement) was used in all operations. Simultaneous bilateral THAs were not included. There were 1,123 patients who had both hips operated on; all operations were performed on a different day and they were included in the study as separate procedures.

### Statistics

Mortality at 2 days, 10 days, 30 days, 90 days, 180 days, and one year was assessed. Binary logistic regression was used to compare the mortality in the cemented HA group compared with the uncemented HA group and the mortality in the hybrid THA and cemented THA groups compared with the uncemented THA group. A random intercept logistic model was used to account for the dependency between operations performed for the same patient. Analyses were adjusted for potential confounding factors age, gender, ASA class, and year of surgery. In addition, subgroup analysis for patients of ASA class IV was applied to compare the mortality between the cemented and the uncemented HA groups. This analysis was also adjusted for age, sex, and year of surgery. P-values less than 0.05 were considered statistically significant. Statistical analyses were done with SAS System for Windows, version 9.4 (SAS Institute Inc., Cary, NC, USA).

### Ethics, funding, and potential conflicts of interest

Ethical approval was granted by the Regional Ethical Review Board in Turku (approval number THL/926/5.05.00/2017). This research received no specific grant from any funding agency in the public, commercial, or not-for-profit sectors. The authors declare no conflicts of interest.

## Results

The number of deaths for each group is presented in Table 2. There were no statistically significant differences in mortality at any time point when comparing hybrid THA with uncemented THA (Table 3). There were no deaths during the 1st 2 days postoperatively in the uncemented THA group, 1 (0.2%) in the hybrid group, and 3 (0.2%) in the cemented group (Table 2). There were more deaths in the cemented THA group when comparing with the uncemented THA group after adjusting the groups for age, sex, ASA class, and year of surgery at 180 days (OR 2.0; CI 1.0–3.7) postoperatively (Table 3). No statistically significant difference was found at other time points.

**Table 2.** Number of deaths

	Uncemented	Cemented	Uncemented	Hybrid	Cemented
	HA	HA	THA	THA	THA
Number of operations	1,173	1,935	5,563	640	1,366
Number of deaths, n (%)					
0–2 days	22 (1.9)	50 (2.6)	0	1 (0.2)	3 (0.2)
0–10 days	61 (5.2)	99 (5.1)	4 (0.1)	2 (0.3)	5 (0.4)
0–30 days	105 (9.0)	171 (8.9)	6 (0.1)	2 (0.3)	9 (0.7)
0–90 days	173 (14.8)	303 (15.7)	17 (0.3)	5 (0.8)	19 (1.4)
0–180 days	227 (19.4)	388 (20.1)	29 (0.5)	8 (0.3)	29 (2.1)
0–365 days	279 (23.8)	503 (26.0)	50 (0.9)	14 (2.2)	41 (3.0)

THA = total hip arthroplasty. HA = hemiarthroplasty.

**Table 3.** Postoperative mortality risk for uncemented THA compared with hybrid and cemented THA, OR, 95% CI, and p-value (adjusted for age, sex, ASA class, and year of surgery)

	Uncemented	Hybrid THA		Cemented THA	
	THA (ref.)	OR (95% CI)	p-value	OR (95% CI)	p-value
0–2 days	1	NA		NA	
0–10 days	1	3.2 (0.5–20.2)	0.2	1.7 (0.3–8.7)	0.5
0–30 days	1	1.7 (0.6–4.9)	0.4	1.6 (0.7–3.6)	0.3
0–90 days	1	1.7 (0.6–4.9)	0.4	1.6 (0.7–3.6)	0.3
0–180 days	1	1.7 (0.7–3.9)	0.2	2.0 (1.0–3.7)	0.04
0–365 days	1	1.5 (0.8–2.9)	0.2	1.6 (0.9–2.7)	0.08

NA = Not available due to zero deaths during the first two days postoperatively in the uncemented THA group.

With unadjusted data there were more deaths in the cemented HA group (50 deaths, 2.6%) when compared with the uncemented HA group (22 deaths, 1.9%) during the 1st 2 days postoperatively (Table 2). Of these patients 30 in the cemented HA group and 10 in the uncemented group were classified as ASA class IV. Age, sex, ASA class, and year of surgery adjusted mortality did not differ statistically significantly between the groups during the 1st 2 postoperative days (OR 1.4; CI 0.8–2.3) (Table 4). In addition, there was no statistically significant difference in the mortality rate between cemented and uncemented HA at any other time point either.

**Table 4.** Postoperative mortality risk for cemented HA compared with uncemented HA, OR, 95% CI, and p-value (adjusted for age, sex, ASA class, and year of surgery)

	Uncemented	Cemented THA	
	THA (ref.)	OR (95% CI)	p-value
0–2 days	1	1.4 (0.8–2.3)	0.3
0–10 days	1	1.0 (0.7–1.4)	1.0
0–30 days	1	1.0 (0.8–1.3)	1.0
0–90 days	1	1.1 (0.9–1.3)	0.5
0–180 days	1	1.0 (0.8–1.2)	0.9
0–365 days	1	1.1 (0.9–1.3)	0.4

In the subgroup analyses of patients of ASA class IV there was a difference in mortality that did not reach statistical significance during the 1st 2 postoperative days in the cemented HA group when compared with the uncemented HA group (OR 2.1; CI 0.9–4.7). No statistically significant difference in mortality was found thereafter either.

## Discussion

We found no statistically significant difference in the adjusted early postoperative mortality after cemented THA compared with uncemented or hybrid THA. Further, we found no statistically significant differences in the adjusted mortality between cemented and uncemented HA at any time point. However, in the subgroup analyses of ASA class IV HA patients there was a difference that did not quite reach our criteria for statistical significance during the 1st 2 days postoperatively in the cemented HA group compared with the uncemented HA group. Based on our results, cementing may be a safe option in both elective and fracture hip surgery. However, in the most fragile HA patient group caution is needed at the moment of cementing.

Cementing is the gold standard for implant fixation, especially in elderly patients treated for femoral neck fractures. Bone cement has been thought to strengthen bone from inside and, therefore, to decrease the risk for periprosthetic fracture, osteolysis, and loosening. All major registries show lower revision rates for cemented implants in elderly patients with OA (Swedish Hip Arthroplasty Register 2013, AOANJRR 2016, NJR 2016, FAR 2017). Additionally, there is evidence that cementing the stem reduces postoperative pain and leads to better mobility (Parker et al. 2010). Cementing may also decrease the risk of reoperation when compared with uncemented hemiarthroplasty in hip fracture patients (Gjertsen et al. 2012, Yli-Kyyny et al. 2014). Due to these data, the proportion of cemented stems has been increasing recently and 62% of the HA patients in this study were cemented. Earlier studies reported that cementing of the hip device was associated with a risk of BCIS increasing perioperative morbidity and mortality (Coventry et al. 1974, Ereth et al. 1992, Parvizi et al. 1999). It has been suggested that the risk of BCIS might be increased in hip fracture patients who are, in general, old and fragile and have several comorbidities (Keating et al. 2006, Moja et al. 2012). Improvements in surgical and anesthesiology techniques and implants, the use of low molecular weight heparins (LMWHs) in the 1980s, and operating room sterility have significantly reduced overall mortality risks associated with hip arthroplasty.

There are earlier studies reporting increased early postoperative mortality in patients treated with cemented HA (Parvizi et al. 1999, Yli-Kyyny et al. 2014). We found a higher proportion of perioperative deaths (0–2 days postoperatively) in the cemented HA group than in the uncemented HA group. It is possible that these numbers include deaths due to BCIS; nonetheless, this could not be confirmed as we did not have access to the cause of death. However, this difference vanished after adjusting data for age, sex, and ASA class, suggesting that the difference was not due to cementing. This is in line with registry studies from Australia and UK where there has not been an increase in early postoperative mortality when comparing cemented and uncemented implants (Costa et al. 2011, Costain et al. 2011). Also, in studies reporting increased early postoperative mortality when using bone cement, the risk disappeared after the first postoperative week or even reversed to a lower mortality for those treated with a cemented prosthesis (Costain et al. 2011, Yli-Kyyny et al. 2014). In our study, in the most fragile patient group (ASA class IV) we found a tendency toward higher mortality during the 1st 2 days when bone cement was used. However, most of these patients survive the immediate postoperative period and long-term outcome may motivate cementation.

We found in the adjusted data an increased risk of death in patients treated with cemented THA when compared with patients treated with uncemented THA at 180 days postoperatively. This late mortality, however, is not explained by BCIS. It is probably due to the baseline differences in the treatment groups: patients treated with cemented THA were older than patients treated with uncemented THA. Our finding is in line with an earlier study that found no increase in mortality with cemented THA compared with uncemented THA during the first 30 postoperative days (Parvizi et al. 2001).

Our study has several limitations. First, we do not have information concerning perioperative resuscitations due to BCIS that did not lead to the patient’s death. It is possible that there is more morbidity due to cementing, which might affect to patient’s quality of life. Second, we did not have the causes of death. Therefore, we do not know the absolute number of deaths due to BCIS. However, we focused on overall mortality. Also, besides ASA class, we did not have information on patients’ comorbidities known to affect the risk of death (such as dementia or congestive heart failure) and therefore study groups could not be adjusted for these. Third, the early mortality rate after THA is low and it is possible that in a larger population some smaller differences in the mortality could be detected. Further, some surgeons may have hesitated to use bone cement due to the possibility of BCIS. This may cause some selection bias to our results, although we think of minor importance.

Lastly, that differences in the mortality rates did not reach statistical significance does not exclude excess mortality in a group, especially when confidence intervals are large. However, our data imply that it is unlikely bone cementing would increase mortality rates in THA patients, although we cannot exclude some excess mortality in HA patients.

In summary, there was no statistically significant difference in adjusted perioperative and short-term postoperative mortality between patients treated with cemented HA or THA and patients treated with uncemented HA or THA or hybrid THA in our material. Cementing may still be a safe option in both elective and fracture hip surgery. Excess mortality of cemented THA and HA in the longer term is comorbidity related, not due to BCIS.  

KM designed and coordinated the study. EE collected the data and drafted the manuscript. IL helped to draft the manuscript. TV calculated the statistics. KM, IL, KI, AL, TV, and EE contributed to the interpretation of the data and results and to the preparation of the manuscript. All authors read the final manuscript.

*Acta* thanks Anne Garland and Sven-Erik Ricksten for help with peer review of this study.

## References

[CIT0001] AOANJRR Annual Report 2016. AOANJRR; 2016.

[CIT0002] Berstock J R, Beswick A D, Lenguerrand E, Whitehouse M R, Blom A W Mortality after total hip replacement surgery: a systematic review. Bone Joint Res 2014; 3(6): 175–82. 24894596 10.1302/2046-3758.36.2000239 PMC4054013

[CIT0003] Buckwalter A E, Callaghan J J, Liu S S, Pedersen D R, Leinen J A, Johnston R C, Goetz D D, Sullivan P M Results of Charnley total hip arthroplasty with use of improved femoral cementing techniques. J Bone Joint Surg Am 2006; 88(7): 1481–5. 16818973 10.2106/JBJS.E.00818

[CIT0004] Costa M L, Griffin X L, Pendleton N, Pearson M, Parsons N Does cementing the femoral component increase the risk of peri-operative mortality for patients having replacement surgery for a fracture of the neck of femur? J Bone Joint Surg Br 2011; 93-B(10): 1405–10. 10.1302/0301-620X.93B10.26690 21969443

[CIT0005] Costain D J, Whitehouse S L, Pratt N L, Graves S E, Ryan P, Crawford R W Perioperative mortality after hemiarthroplasty related to fixation method. Acta Orthop 2011; 82(3): 275–81. 21561308 10.3109/17453674.2011.584208 PMC3235304

[CIT0006] Coventry M B, Beckenbaugh R D, Nolan D R, Ilstrup D M 2,012 total hip arthroplasties: a study of postoperative course and early complications. J Bone Joint Surg Am 1974; 56(2): 273–84. 4452686

[CIT0007] Current Care Guidelines Hip fracture. http://www.kaypahoito.fi/web/kh/suositukset/suositus?id=hoi50040

[CIT0008] Donaldson A J, Thomson H E, Harper N J, Kenny N W Bone cement implantation syndrome. Br J Anaesth 2009; 102(1): 12–22. 19059919 10.1093/bja/aen328

[CIT0009] Ereth M H, Weber J G, Abel M D, Lennon R L, Lewallen D G, Ilstrup D M, Rehder K Cemented versus noncemented total hip arthroplasty: embolism, hemodynamics, and intrapulmonary shunting. Mayo Clin Proc 1992; 67(11): 1066–74. 1434866 10.1016/s0025-6196(12)61121-5

[CIT0010] Finnish Arthroplasty Register (FAR) http://www.thl.fi/far; 2017.

[CIT0011] Gjertsen J-E, Lie S A, Vinje T, Engesaeter L B, Hallan G, Matre K, Furnes O More re-operations after uncemented than cemented hemiarthroplasty used in the treatment of displaced fractures of the femoral neck: an observational study of 11,116 hemiarthroplasties from a national register. J Bone Joint Surg Br 2012; 94(8): 1113–19. 22844055 10.1302/0301-620X.94B8.29155

[CIT0012] Hunt L P, Ben-Shlomo Y, Clark E M, Dieppe P, Judge A, MacGregor A J, Tobias J H, Vernon K, Blom A W 90-day mortality after 409 096 total hip replacements for osteoarthritis, from the National Joint Registry for England and Wales: a retrospective analysis. Lancet 2013; 382(9898): 1097–104. 24075049 10.1016/S0140-6736(13)61749-3

[CIT0013] Issack P S, Botero H G, Hiebert R N, Bong M R, Stuchin S A, Zuckerman J D, Di Cesare P E Sixteen-year follow-up of the cemented spectron femoral stem for hip arthroplasty. J Arthroplasty 2003; 18(7): 925–30. 14566751 10.1016/s0883-5403(03)00336-x

[CIT0014] Keating J F, Grant A, Masson M, Scott N W, Forbes J F F, Forbes J F F Randomized comparison of reduction and fixation, bipolar hemiarthroplasty, and total hip arthroplasty: treatment of displaced intracapsular hip fractures in healthy older patients. J Bone Joint Surg Am 2006; 88(2): 249–60. 16452734 10.2106/JBJS.E.00215

[CIT0015] Moja L, Piatti A, Pecoraro V, Ricci C, Virgili G, Salanti G, Germagnoli L, Liberati A, Banfi G Timing matters in hip fracture surgery: patients operated within 48 hours have better outcomes. A meta-analysis and meta-regression of over 190,000 patients. PLoS One 2012; 7(10): e46175. 23056256 10.1371/journal.pone.0046175 PMC3463569

[CIT0016] Morshed S, Bozic K J, Ries M D, Malchau H, Colford J M Comparison of cemented and uncemented fixation in total hip replacement: a meta-analysis. Acta Orthop 2007; 78(3): 315–26. 17611843 10.1080/17453670710013861

[CIT0017] Mäkelä K T, Matilainen M, Pulkkinen P, Fenstad A M, Havelin L, Engesaeter L, Furnes O, Pedersen A B, Overgaard S, Kärrholm J, Malchau H, Garellick G, Ranstam J, Eskelinen A Failure rate of cemented and uncemented total hip replacements: register study of combined Nordic database of four nations. BMJ 2014; 348(January): f7592. 24418635 10.1136/bmj.f7592

[CIT0018] National Clinical Guideline C National Institute for Health and Clinical Excellence: Guidance. Manag Hip Fract Adults 2011. London: NIHCE; 2011.

[CIT0019] NJR NJR 13th Annual Report. Hemel Hempstead: National Joint Registry; 2016.

[CIT0020] Parker M J, Gurusamy K S, Azegami S Arthroplasties (with and without bone cement) for proximal femoral fractures in adults. Cochrane Database Syst Rev 2010; (6): CD001706. 20556753 10.1002/14651858.CD001706.pub4 PMC12360749

[CIT0021] Parvizi J, Holiday A D, Ereth M H, Lewallen D G The Frank Stinchfield Award. Sudden death during primary hip arthroplasty. Clin Orthop Relat Res 1999; (369): 39–48. 10.1097/00003086-199912000-00005 10611859

[CIT0022] Parvizi J, Johnson B G, Rowland C, Ereth M H, Lewallen DG Thirty-day mortality after elective total hip arthroplasty. J Bone Joint Surg Am 2001; 83-A(10): 1524–8. 10.2106/00004623-200110000-00010 11679603

[CIT0023] Rogmark C, Fenstad A M, Leonardsson O, Engesaeter L B, Kärrholm J, Furnes O, Garellick G, Gjertsen J-E Posterior approach and uncemented stems increases the risk of reoperation after hemiarthroplasties in elderly hip fracture patients. Acta Orthop 2014; 85(1): 18–25. 24460108 10.3109/17453674.2014.885356 PMC3940987

[CIT0024] Smith T, Pelpola K, Ball M, Ong A, Myint P K Pre-operative indicators for mortality following hip fracture surgery: a systematic review and meta-analysis. Age Ageing 2014; 43(4): 464–71. 24895018 10.1093/ageing/afu065

[CIT0025] Swedish Hip Arthroplasty Register Annual Report; 2013.

[CIT0026] Yli-Kyyny T, Sund R, Heinänen M, Venesmaa P, Kröger H Cemented or uncemented hemiarthroplasty for the treatment of femoral neck fractures? Acta Orthop 2014; 85(1): 49–53. 24397746 10.3109/17453674.2013.878827 PMC3940991

